# Epidemiological trends of malaria infection in Jeddah, Saudi Arabia, 2018-2023

**DOI:** 10.3389/fpubh.2024.1476951

**Published:** 2024-11-27

**Authors:** Rana Alghamdi, Ahmed Bedaiwi, Ashwaq M. Al-Nazawi

**Affiliations:** ^1^Vector-Borne and Zoonotic Diseases Administration, Public Health, Second Health Cluster, Jeddah, Saudi Arabia; ^2^Department of Vector-Borne and Zoonotic Diseases, Public Health Programs, Ministry of Health, Jeddah, Saudi Arabia; ^3^Department of Public Health, College of Nursing and Health Sciences, Jazan University, Jazan, Saudi Arabia; ^4^Laboratory Department, Jazan University Hospital, Jazan University, Jazan, Saudi Arabia

**Keywords:** malaria, Anopheles, mosquitoes, Saudi Arabia, plasmodium

## Abstract

**Background:**

Malaria poses a significant global public health challenge, especially in tropical regions. Saudi Arabia established the malaria elimination program decades ago, and implemented public health strategies to reduce malaria burden. Every year, Saudi Arabia welcomes millions of people worldwide, particularly from endemic countries, for work, religious activities, visits, and tourism. Jeddah city plays a vital role as a logistical center for the entry of travelers through its airports and seaports. Gaining insight into the demographic characteristics of malaria cases in Jeddah governorate is a crucial assessment for public health measures to reduce the malaria burden and support the malaria elimination program.

**Method:**

In this cross-sectional study, we described the characteristics of malaria cases reported by the Vector-Borne and Zoonotic Diseases Administration in Jeddah from 2018 to 2023. We also conducted a descriptive analysis using R and QGIS software to comprehend the epidemiological status of malaria cases in the Jeddah governorate.

**Results:**

A total of 2,124 cases were reported from 2018 to 2023. Pakistanis are considered the major nationality with malaria by 47.56%. African countries such as Sudan, Ethiopia, Nigeria, and Chad showed more than a third of malaria cases reported. Plasmodium Vivax and Falciparum were reported by almost 57 and 38%, respectively. An increasing number of malaria cases observed every year, except during the COVID-19 pandemic.

**Conclusion:**

This study illustrated the epidemiology trend of malaria cases reported in Jeddah city between 2018 and 2023. Its findings highlighted the importance of strengthened emphasis on malaria prevention protocols in the Kingdom of Saudi Arabia.

## Introduction

Malaria is a serious global public health problem worldwide, especially in tropical regions. It is an acute febrile disease caused by a blood protozoa called the malaria parasite belonging to Plasmodium spp. (1). The Plasmodium parasites that typically infect humans include P.falciparum (PF), P.vivax (PV), P.ovale (PO), P.malariae (PM) and P.knowlesi (PK) (2). Among these, PF is the most dangerous (1). Plasmodium species spread primarily through the bites of infected female Anopheles mosquitoes, which breed in stagnant water sources under specific environmental conditions, such as warm temperatures (1). Symptoms of malaria are fever accompanied by chill and flu-like symptoms (1). In regions where malaria transmission is endemic, many people carry malaria parasites asymptomatically as a result of naturally acquired immunity (3). If malaria is left untreated, it could lead to serious health complications and death (1). Diagnosing the disease is often achieved by microscopic examination of blood samples or rapid tests of blood antigens (1). Many studies indicated that the prevalence of malaria infection increased after 2015 (4). Malaria has infected nearly 219 million people and caused 435,000 deaths in 2017 internationally (4). In 2021, malaria killed 619 million people around the world, and the African region disproportionately accounted for 95% of the deaths of global malaria cases (1). Yet, cases of malaria are still reported in non-endemic countries, often brought by travelers from endemic regions (5).

Historically, the Kingdom of Saudi Arabia has faced malaria challenges since the late 1940s (6). Saudi Arabia recorded two epidemic cycles in the 1950s and the 1990s. The first malaria epidemic was in Jeddah between December 1950 and February 1951; it recorded 3,717 cases in ten weeks due to Hajj (6). In the second cycle in 1992, the Al-Hassa region re-emerged locally acquired PV infections, although it was declared free of malaria in the 1970s (6). Two significant epidemics followed this in 1994 and 1995 before the region could completely eliminate the disease (6). Around the same time, other outbreaks of over 400 PF malaria cases were reported in the southwestern areas, especially during the El Nino rains in 1998, leading to epidemics with over 31,000 cases reported in the Jazan region (6).

The Kingdom of Saudi Arabia is located in the southwest corner of Asia and extends over 2,150,000 square kilometers, dominating about four-fifths of the Arabian Peninsula (7). It is bordered by the Red Sea on the West side, by the Arabian Gulf and the United Arab Emirates and Qatar on the East side, by Yemen and Oman on the South side, and finally by Jordan, Iraq, and Kuwait on the North side. The Kingdom has a diverse topography because of its large area (7). Most terrain consists of desert, arid desert, lowland, steppe, forest, and mountains (7). The southern provinces of Jazan and Asir have mountainous and valley areas with seasonal rainfall, which create ideal breeding conditions for Anopheles (6). The highest vector density in these areas is during the rainy season, aligning with malaria transmission peaks (8). The prevailing malaria species in Saudi Arabia is PF (4, 5, 9). Malaria vectors present in Saudi Arabia are Anopheles Arabiensis, Sergentii, Stephensi, Superpictus, and Culicifacies (2). The incidence of malaria in Saudi Arabia is primarily concentrated along the southern regions of the Red Sea coast, extending to the border with Yemen (10).

Efforts to control and eliminate malaria in Saudi Arabia have been ongoing for several decades. In 1948, Saudi Arabia established the National Malaria Control Program, targeting heavily impacted regions with malaria (10). The Arabian American Oil Company (ARAMCO) initiated a malaria control program in the Eastern province in 1948, mainly to protect employees living around the oases through water management techniques and health education (11, 12). Also, the Saudi government adopted the program in 1952 to implement preventive strategies to malarious districts and protect pilgrims in Mecca and Medina (11). Furthermore, Saudi Arabia joined the global malaria elimination program in 1963, and by the early 1970s, disease transmission had been interrupted in the Eastern and Northern provinces (11). Since the introduction of malaria elimination programs, Saudi Arabia has made significant progress in reducing the burden and geographic extent of malaria nationwide. Despite all this success, there is still a limited number of remaining malaria foci in the Jazan and Aseer regions (6, 10).

The population movement is an essential factor that has halted the progress against the malaria epidemic in Saudi Arabia. For instance, Saudi Arabia receives Hajj pilgrims worldwide every year. Moreover, there was a massive cross-border migration of people fleeing civil war in Yemen (6). The Yemen crisis contributed to suspending malaria control operations at the Saudi border, where the remaining malaria vector is present (6). Most cases reported in Saudi Arabia were visitors and foreign workers from other endemic countries; only about 5% were locals from the Jazan and Aseer regions (6). Increased air travel from endemic regions can heighten the risk of malaria re-emerging or resurging in areas that were previously malaria-free or had low transmission rates (13). A study indicates that mosquitoes have adapted to the growth of international travel and climate change, contributing to a rise in malaria transmission around airports; this locally acquired variant is called “Airport malaria” (14). In passenger luggage, cargo, or sea transportation, infected tropical mosquitoes can escape the failed disinfection and survive (14). Therefore, continued efforts are needed to prevent malaria re-introduction into previously malaria-free regions through airports and seaports (15).

Jeddah city is a coastal city characterized by limited freshwater sources and desert climate, which renders it unsuitable for Anopheles mosquitoes (16). The primary vector-borne disease risk in Jeddah is associated with Aedes species, which transmits dengue fever rather than malaria (17). Jeddah is a key logistical hub for incoming travelers via airports and seaports. Every year, Jeddah receives Hajj pilgrims from around the world, especially from prevalent malaria countries, such as Pakistan, Yemen, Ethiopia, and Sudan (10). During the political crisis in Sudan 2023, Jeddah welcomed people from Sudan (18). Due to the challenges of population movement across the border, the incidence rate of malaria is increasing in Jeddah. This research paper provides a comprehensive descriptive analysis of malaria cases recorded in the Jeddah governorate from 2018 to 2023, aiming to support the malaria elimination program and develop strategies to prevent its reintroduction.

## Methodology

### Sample collection, case confirmation, and statistics

We conducted a cross-sectional study and analyzed 6 years of malaria data collected between 2018 and 2023 from the Ministry of Health -Vector-Borne and Zoonotic Diseases (VBZD) administration in Jeddah, Saudi Arabia. The VBZD receives notifications of malaria cases from healthcare facilities in Jeddah after they have been identified and documented. VBZD’s practitioners perform procedures in conducting epidemiological investigations to gather patient history and identify potential risk factors. Additionally, they ensure that healthcare facilities collect blood samples and test them in the reference laboratory in Jeddah for further confirmation and differentiation of plasmodium species diagnosis. Malaria microscopy is the gold standard test to confirm the type of malaria species. The data was filtered based on epidemiological weeks. The total number of malaria cases was 2,128. Four cases were excluded due to missing age and gender information. The final total number was 2,124 malaria cases. We performed descriptive analysis using R software to measure the frequencies and display the demographic characteristics of malaria cases. We formed a QGIS map displaying the infection’s origin, and created graphs and tables based on different variables.

### Covariates

We examined the following demographic variables from VBZD 2018–2023: number of malaria cases by year (2018–2023), age included as groups (<20, 20–29, 30–39, 40–49, 50–59 and ≥ 60), sex (male or female), nationalities, plasmodium species, and site of infection. We included the sites of infection that recorded 10 or more malaria cases in the 6 years to address misreporting in non-epidemic malaria countries.

### Ethics statement

We received IRB approval from the Ministry of Health. The data used in this paper are derived from routine data collection by the Ministry of Health—VBZD Administration in Jeddah. Patients’ information has been anonymized. The raw data collected is not subject to ethical review as they form part of routine analysis of malaria reported cases in the Makkah region.

## Results

This study included 2,124 malaria cases, with approximately 45% identified in 2023. The mean age for malaria cases was 33 years, 69% of cases were between 20 to 40 years old. We found that men are more likely to have malaria than women by 87 and 13%, respectively. The data showed that the ratio of confirmed cases from Saudi to non-Saudi was 1.4 to 98.6%, respectively. The majority of malaria cases were Pakistanis (47.56%), Sudanese (19.15%), Yemenis (9.34%), Ethiopians (9.20%), Indians (3.11%), Chadians (2.22%), Nigerians (1.98%), and others (7.41%) (Table 1).

**Table 1:** Demographic characteristics of malaria cases, VBZD Jeddah 2018–2023.

Participants characteristics	No. (column %) (*n* = 2,124)
Years	
2018	201 (9.46)
2019	249 (11.72)
2020	144 (6.78)
2021	155 (7.30)
2022	410 (19.30)
2023	965 (45.43)
Age groups	
< 20	154 (7.25)
20–29	880 (41.43)
30–39	591 (27.82)
40–49	244 (11.49)
50–59	151 (7.11)
≥ 60	104 (4.90)
Sex	
Male	1,847 (86.96)
Female	277 (13.04)
Nationalities	
Pakistan	1,008 (47.56)
Sudan	406 (19.15)
Yemen	198 (9.34)
Ethiopia	195 (9.20)
India	66 (3.11)
Chad	47 (2.22)
Nigeria	42 (1.98)
Saudi Arabia	30 (1.42)
Othersᵃ	127 (5.99)
Missing	5
ᵃOther nationalities <20 cases per each nationality	

**Figure 1:**
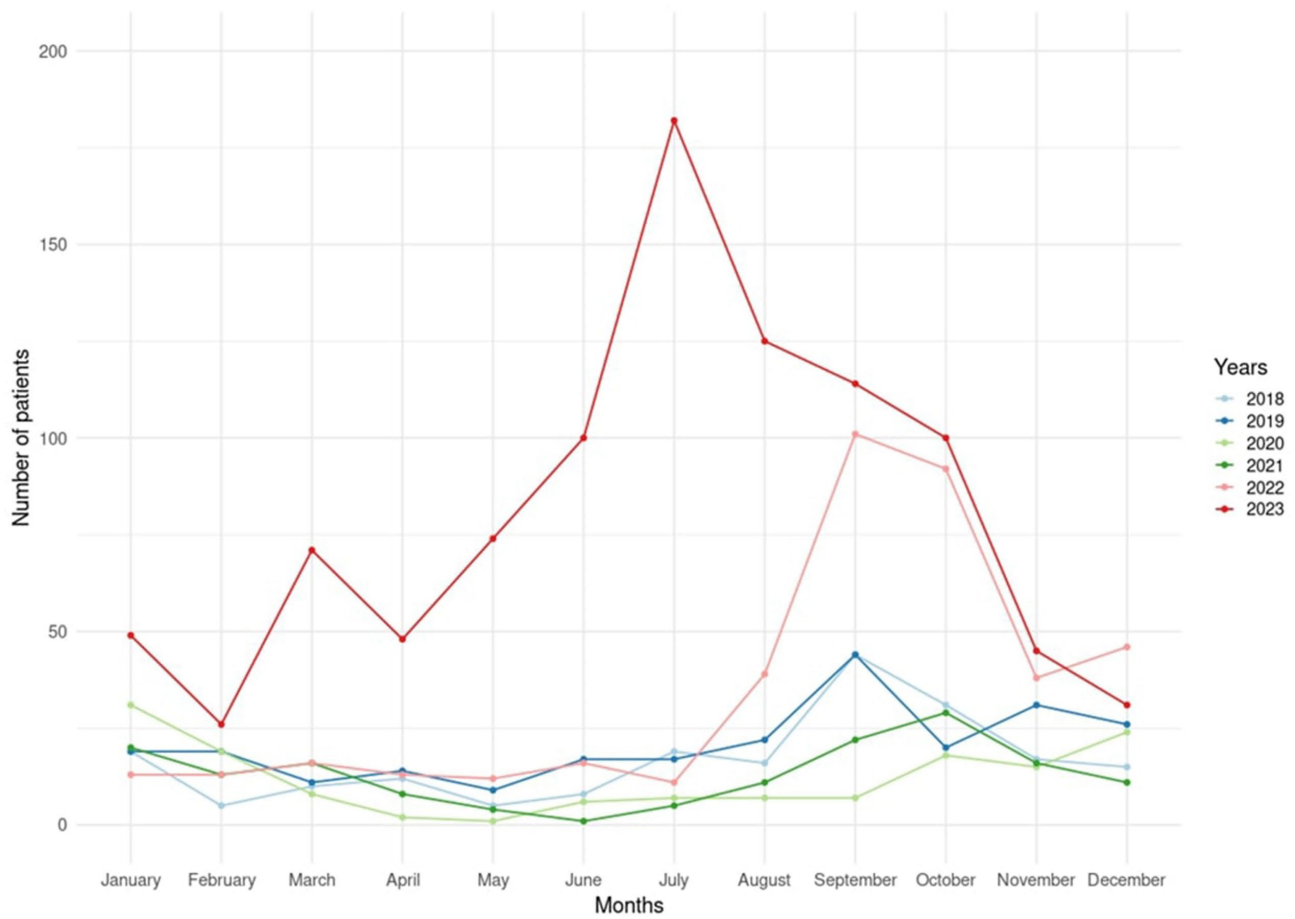
Number of malaria cases per month reported by VBZD Jeddah between 2018 and 2023.

In Figure 1, the chart illustrates the number of malaria cases per month in 6 years. From 2018 until 2022, a notable trend shows an increase in malaria cases during the second half of each year. However, in the first quarter (Q1), including Jan, Feb, and March, of 2023, the data presented an early rise of 146 malaria cases compared to the Q1 from 2018 to 2022 by 34, 49, 58, 49, and 42 cases, respectively. In July 2023, reported malaria cases hit the highest monthly total, reaching a peak over the past 6 years. This peak represented more than 15-fold of reported cases by 182 cases compared to the average from 2018 to 2022 (12 cases) in July. Furthermore, the line chart presents a fluctuation of malaria cases at the Q1 and the beginning of Q2 in 2023. Based on evidence of non-existence of malaria vectors in Jeddah (6), this inconsistency of malaria cases in the first half of 2023 might happen due to various reasons, such as surveillance systems challenges during annual vacations in healthcare facilities, a misdiagnosis due to co-morbidity with dengue fever, or an elevated imported travelers from malaria epidemic countries.

**Figure 2:**
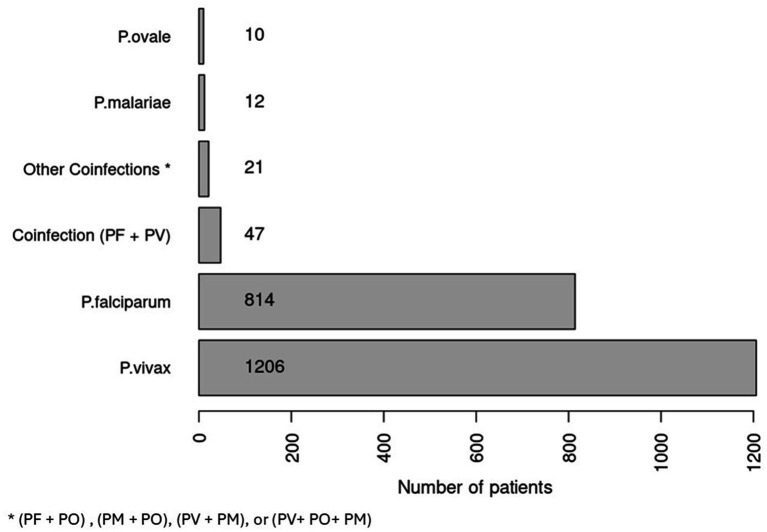
Number of patients based on plasmodium species by VBZD Jeddah between 2018 and 2023.

Among 2,124 Malaria cases, we excluded 14 cases with no confirmatory plasmodium species result. The PV infected more than half of cases (57.15%). The PF presented 38.58% of cases. We found that mixed infections of PV and PF accounted for 2.23%, while other co-infections of PV, PO, and PM comprised 1%, with all these cases imported (Figure 2).

**Figure 3:**
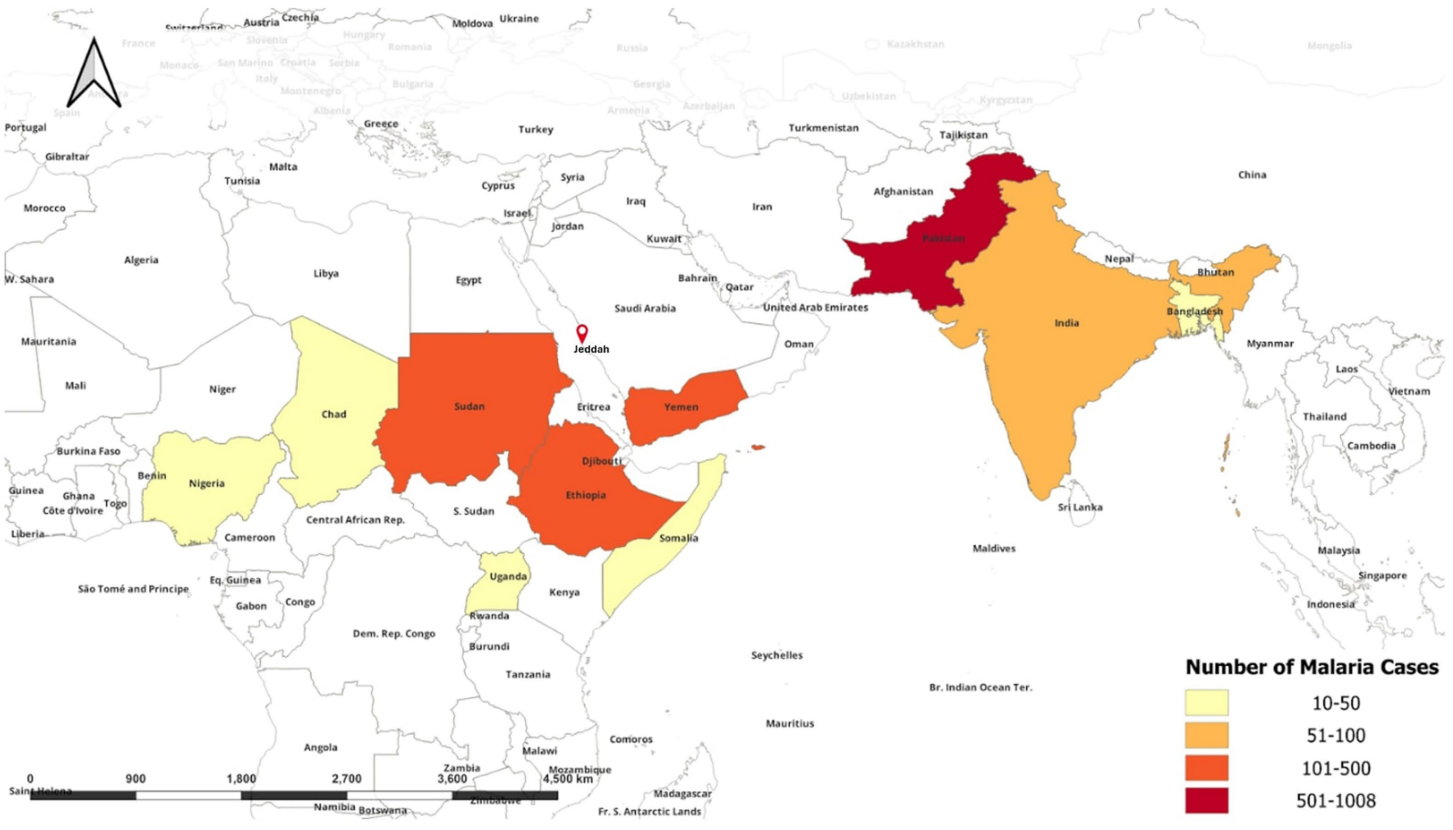
A world map showing the origin of recorded malaria cases in Jeddah from 2018 to 2023.

Understanding the primary infection sites for malaria cases imported to Jeddah is crucial for effective public health prevention strategies. By pinpointing where infections originate, authorities can implement targeted public health measures. This approach helps in reducing transmission and ensuring treatment of malaria cases. Only 2,073 malaria cases had information about the origin of infection. Figure 3 shows the number of malaria cases per site of infection. The data reveals that Pakistan accounts for the majority of cases (47.56%) of malaria infections. African nations such as Sudan, Ethiopia, Chad, Nigeria, Somalia, and Uganda collectively contribute to nearly 37% of the reported cases. Additionally, Asian countries, including Yemen, India, and Bangladesh, represent 13% of the total malaria cases documented.

## Discussion

From 2022 to 2023, the number of malaria cases in Jeddah Governorate surged to more than double. There is a noticeable trend of increasing malaria cases yearly, with exceptions in 2020 and 2021 due to travel restrictions imposed by COVID-19. The strict travel restrictions due to the COVID-19 pandemic have decreased population movement and influenced malaria epidemic internationally (19). One of the reasons for the sudden increase was that the Saudi Arabia allowed Umrah throughout the year except for the month of Hajj in early 2023. Moreover, the Ministry of Tourism announced a 37% increase in tourists in 2023 compared to the first half of 2022 (20). Besides, the Ministry of Foreign Affairs in Saudi Arabia evacuated more than 8,000 people from Sudan in May 2023 due to the political and security crisis that led to the civil war (18). These regulatory restrictions in the region may have led to increased travel from endemic countries such as Pakistan, Sudan, India, Yemen, and various African nations, which may have raised reported malaria cases.

We strongly advise that malaria testing, including rapid or microscopic tests, be mandatory for visa applicants from endemic countries for leisure, work, or religious purposes. The test serves various purposes, such as checking for infectious diseases or ensuring the applicant meets health requirements. Additionally, it would enable the public health department to track malaria-infected travelers upon arriving in the Kingdom, ensuring patients adhere to their treatment plans. Many countries require migrant workers to undergo a medical examination upon arrival and subsequent periodic check-ups to maintain their employment and residency status (21). Therefore, one reliable way to support the malaria elimination program is to incorporate malaria health assessment requirements into visa applications.

Awareness plays a major role in malaria prevention and elimination (22). Travelers are most vulnerable to malaria (1); from this study, Saudi nationals accounted for only 30 of all malaria cases, primarily due to travel to endemic countries. While the majority of cases (80%) occur among individuals aged 20 to 50 years. This age group, mainly composed of non-permanent residents, travels both within the Kingdom and abroad for work, religion, and vacation purposes. Enhancing travelers’ awareness of malaria prevention in endemic countries is advisable by emphasizing the importance of avoiding mosquito bites and taking preventive medications (1, 22, 23).

The distribution of Plasmodium species reported from Jeddah Governorate showed PV was the highest, consistent with global distribution patterns (24, 25). The current study shows PF is the second most frequently diagnosed malaria species. These findings have been reported previously in Saudi Arabia (26). Conversely, a study has reported that PF was dominant over PV in southwestern region of Saudi Arabia (10).

The data indicated that the majority of malaria cases are among Pakistanis, which correlates with a four-fold increase in cases in 2023 attributed to flooding in Pakistan in 2022, which affected approximately 1.6 million people (27). Additionally, travel plays an important role in malaria transmission dynamics. Increased international flights are also associated with many malaria cases (14). Air travel could transport infected vectors from endemic to non-endemic countries “Malaria airport,” potentially facilitating the spread of malaria in the nearby working and living neighbors (28). Therefore, intensifying in-flight disinfection and implementing appropriate precautions for countries known to be that endemic malaria is important, especially after recent floods to prevent the establishment of a local reservoir (14, 28, 29). A robust mosquito control program is mandated to avoid possible failure of disinfection.

### Limitations

Our study had several limitations:

1. We believe there are missing malaria cases due to asymptomatic patients or misdiagnosis of cases with similar disease symptoms, such as flu and dengue fever.
2. Incorrect patient statements regarding the travel history or infection site could affect the accuracy of the epidemiological investigation conducted.
3. We had limited data regarding socioeconomic status and education level to study the association to better identify the population at high risk and need awareness.
4. Data regarding the possibility of developing insecticide resistance is limited.

## Conclusion

The persistent rise in imported malaria cases in Jeddah signifies the critical need for robust public health actions to effectively monitor the issue. The number of malaria cases in 2023 increased by two-fold compared to 2022. The study highlighted the nationalities and predominant species between 2018 and 2023. Most malaria cases reported by VBZD Jeddah were from Pakistan (48.62%), Sudan (19.39%), Ethiopia (9.26%), Yemen (9.26%), and India (3.09%). PV is the predominant species, reported by 57.15%, followed by PF (38.58%). Despite the extensive initiatives undertaken by Saudi health authorities to eradicate malaria, the presence of cases among imported travelers remains a concern. It is suggested that travelers from other countries be checked/diagnosed for malaria parasites by an appropriate and accurate diagnostic method in their home airport before checking in at the airport with an authenticated certificate. Additionally, we advocate for including a malaria test as a prerequisite for issuing visas to enter the Kingdom of Saudi Arabia, alongside a strengthened emphasis on malaria prevention protocols to ensure the continued success of malaria elimination efforts in Saudi Arabia.

## Data Availability

The data analyzed in this study is subject to the following licenses/restrictions: Patients’ information. Requests to access these datasets should be directed to Ahmed Bedaiwi, akbedaiwi@moh.gov.sa.
